# White Matter Changes in Bipolar Disorder, Alzheimer Disease, and Mild Cognitive Impairment: New Insights from DTI

**DOI:** 10.4061/2011/286564

**Published:** 2011-12-01

**Authors:** Aikaterini Xekardaki, Panteleimon Giannakopoulos, Sven Haller

**Affiliations:** ^1^Division of Mental Health and Psychiatry, Department of General Psychiatry, University Hospitals of Geneva, Chemin du Petit-Bel-Air, Geneva, Switzerland; ^2^Division of Old Age Psychiatry (PG), University of Lausanne School of Medicine, Lausanne, Switzerland; ^3^Service Neuro-Diagnostique et Neuro-Interventionnel DISIM, University Hospitals of Geneva, Switzerland

## Abstract

Neuropathological and neuroimaging studies have reported significant changes in white matter in psychiatric and neurodegenerative diseases. Diffusion tensor imaging (DTI), a recently developed technique, enables the detection of microstructural changes in white matter. It is a noninvasive *in vivo* technique that assesses water molecules' diffusion in brain tissues. The most commonly used parameters are axial and radial diffusivity reflecting diffusion along and perpendicular to the axons, as well as mean diffusivity and fractional anisotropy representing global diffusion. Although the combination of these parameters provides valuable information about the integrity of brain circuits, their physiological meaning still remains controversial. After reviewing the basic principles of DTI, we report on recent contributions that used this technique to explore subtle structural changes in white matter occurring in elderly patients with bipolar disorder and Alzheimer disease.

## 1. Introduction

White matter (WM) comprises 40–50% of the adult human brain. At a macroscopical level, it consists of collections of tightly wrapped axons that connect different brain regions. At a biochemical level, WM is mainly formed by myelin, a multilayer sheath of proteins (30%) and lipids (70%) around the axons. Small unwrapped regions called Ranvier nodes serve to mediate the salutatory conduction of the electrical impulse and enhance the velocity of conduction [1]. Neuropathological studies [2] indicate that myelination continues until at least the third decade of life. Other scientists found that WM increases in a roughly linear way until at least the age of 20 [3]. *In vivo* studies using volumetric imaging showed that WM volume increases in frontal and temporal lobes by the fourth decade of life [4, 5] and then steadily decreases. A neuropathological study found WM volume reduction by 28% as a function of age [6]. Interestingly, an assessment of WM volume in piano players suggests that practicing induces plasticity in early age when fiber tracts are still under maturation [7]. The development of modern MR techniques allowed for documenting WM changes in several neurological and psychiatric entities. In this paper, we describe a recent *in vivo* noninvasive technique for WM imaging referred to as diffusion tensor imaging (DTI) and explore its relevance in bipolar disorder and Alzheimer disease. The goal of this paper is to develop the different parameters obtained with DTI, the principal analysis methods, as well as the correlation of DTI-derived parameters with clinical and neuropathological findings in human brains and animal models. We will refer to the application of DTI on Alzheimer disease (a neurodegenerative disease with well-established associated morphological abnormalities in particular in the hippocampal region) and bipolar disorder (a psychiatric disease with currently disputed associated morphological abnormalities) for detection of white matter changes. The choice of these two entities is related to our previous and actual work in these fields of research and our recent work using DTI in bipolar disorder [8]. We included articles analyzing the basic principles of the technique, its correlation to neuropathology as exposed from animal models and human brain banks. DTI, white matter changes, bipolar disorder, AD, and mild cognitive impairment were the key words used to search for articles of DTI applications on these medical entities in Pubmed. In the discussion part, our aim is to provide authors with a critical review of advantages and disadvantages of the DTI technique and its different ways of data analysis, as well as future directions of improvement.

## 2. Diffusion Tensor Imaging

**Figure 1:**
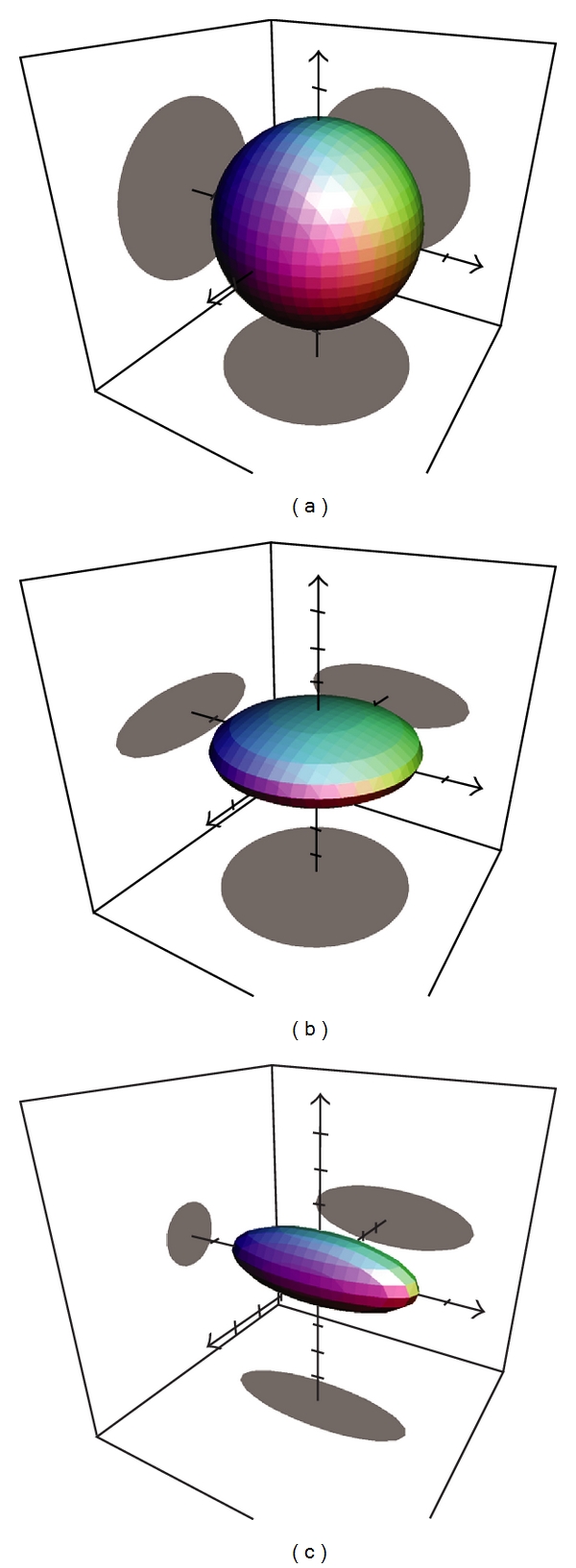
This illustrates the basic tensor shapes of diffusion tensor imaging (DTI). If the diffusion is not restricted, the resulting tensor is a sphere (a). If the diffusion is restricted in only one direction, the resulting tensor is lens-shaped (b). If the diffusion is restricted in two directions, the resulting tensor is cigar-shaped (c).

Diffusion tensor imaging is a promising technique that evaluates *in vivo* brain structure, especially white matter integrity. DTI was originally presented in 1994 [9, 10] and takes advantage of the fact that MR images are essentially sensitive to water protons. Molecular diffusion refers to the Brownian random motion resulting from the thermal energy carried by these molecules [11]. Diffusion is a three-dimensional process. Water is the most convenient molecule to study with MRI. Water proton displacement depends on surrounding tissue microstructure. In cerebrospinal fluid (CSF), water molecules move equally in all directions in space and the resulting diffusion tensor is isotropic. In contrast, diffusion is anisotropic in white matter. Water follows a path along the white matter fiber that is constrained by barriers such as the myelin sheath causing movement to be greater along the long axis of the fiber than perpendicular. Thus, axial diffusion along the fiber is greater than radial diffusion across the fiber [12, 13]. The degree of anisotropy can be expressed by the fractional anisotropy (FA). This is an absolute value that ranges from zero (diffusion equal in all directions resulting into a spherical diffusion tensor, see Figure 1) to 1 (diffusion only in one direction yet zero in the other orthogonal directions resulting in a cigar-shaped tensor of unlimited length). To quantify anisotropic diffusion, the computation of a tensor is required based on data from at least six or more noncollinear gradient directions. The diffusion tensor is a three-dimensional ellipsoid depicting the magnitude and orientation of diffusion in an individual voxel (Figure 1). The ellipsoid has three axes called eigenvectors corresponding to the three orientations of the tensor, and their lengths are called eigenvalues. The longest eigenvalue pointing along the axon direction is called *λ*1 or axial diffusivity, and the two small axes orthogonal to the long one are called *λ*2 and *λ*3. By using the diffusion tensor imaging model we assess the following parameters.

### 2.1. Axial and Radial Diffusivities

The diffusivity parallel to the principal axis of the axon within a voxel of interest is called longitudinal or axial diffusivity or *λ*1 [10]. Radial diffusivity represents diffusivity perpendicular to the first eigenvector: *λτ*: (*λ*2 + *λ*3)/2. Song et al. used a mammalian model (shiverer mouse) that has incomplete myelin formation without any signs of axonal damage or inflammation to find direct associations of directional diffusivity changes with pathological findings [13]. Radial diffusivity increased significantly in these otherwise intact axons reflecting the freer movement of water molecules related to the reduction of myelin. No change of axial diffusivity was reported, suggesting that radial diffusivity could serve as a biomarker of myelin loss or damage. The same group of scientists used a mouse model of retinal ischemia that provokes axonal degeneration in optic nerve and correlated DTI and pathological findings [14]. They found a significant decrease of axial diffusivity, but not radial diffusivity, 3 days after ischemia coinciding with detectable axonal degeneration but with no demyelination. An increase of both axonal and radial diffusivity was reported on the fifth day associated with myelin degeneration at this time. Thus, axial diffusivity is thought to correspond to axonal damage and radial diffusivity to myelin damage. These findings were further confirmed by more recent studies [15, 16], yet basic research is still needed in this domain.

### 2.2. Mean Diffusivity

Mean diffusivity represents the average magnitude of a tensor's water diffusion and is equal to the average of the three eigenvalues (*λ*1 + *λ*2 + *λ*3)/3. Mean diffusivity is the mean molecular motion in a certain voxel, but it provides no elements regarding diffusion directionality. To date, the physiological correlates of this DTI parameter are not well understood.

### 2.3. Fractional Anisotropy

The most commonly used DTI parameter assessed in brain research is fractional anisotropy (FA) [11]. Fractional anisotropy represents the normalized standard deviation of the three diffusivities and is thought to be a marker of WM integrity. In CSF, where diffusivity is equal in all directions, the FA index is zero. In WM, FA increases, showing fast diffusivity along the fibers and a slow diffusivity perpendicular to them. The microstructural changes corresponding to anisotropy changes in WM tissues still remain quite unclear. Decreased FA has been described in tissues with demyelination, edema, gliosis, and inflammation. Myelin is a characteristic anatomical feature of white matter and is thought to play a crucial role in DTI signal. Beaulieu and Allen reported that anisotropy is observed in nonmyelinated fibers as well. The water diffusion of nonmyelinated olfactory nerve was similar to myelinated trigeminal one in garfish [17, 18]. Wimberger et al. revealed anisotropic water diffusion in pup rats in not myelinated tissues [19]. Gulani et al. compared diffusion anisotropy of myelin deficient rats and age-matched controls. They concluded that myelin is not a required factor for the presence of diffusion anisotropy (consistent with the above studies), but its presence plays a major role in diffusion anisotropy generation in WM [20].

## 3. Types of DTI Data Analyses

We will summarize most commonly used methods of DTI data analysis.****

### 3.1. ROI Analysis of Diffusivity Values (e.g., FA or MD)

Region of interest analysis consisting of manually designed or template-based comparison of specific regions among different subjects. They are used in order to calculate and compare DTI parameters in a specific region implicated in a disease. The advantage of this method is the simplicity and the high sensitivity because multiple voxels within a given ROI are averaged thereby enhancing the signal to noise ratio. The disadvantages of this method are that it is time consuming and operator-dependent when regions of interest are drawn manually. Furthermore, as the ROI is designed manually, it does not necessarily correspond to the anatomic borders of a given area. Voxels with significant differences can be averaged with other voxels without differences, masking their effect.

### 3.2. White Matter Tractography

#### 3.2.1. Deterministic Tractography

**Figure 2:**
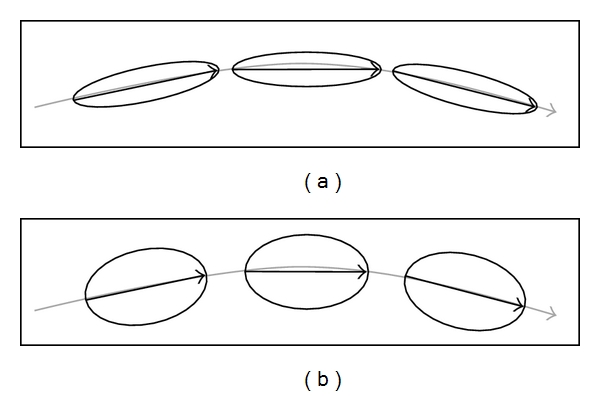
Schematic illustration of deterministic tractography, in a normal subject (a), three adjacent voxels have a clearly directed primary diffusion direction (longitudinal diffusion) indicated as ellipsoidal tensor. A deterministic tractography analysis would result in the indicated tract. Another subject, for example, a patient with a neurodegenerative disease (b) might have a reduction of the directivity of diffusion, evident as less ellipsoidal and more spherical tensors. The direction of the principal direction is however unchanged. This explains why a deterministic tractography analysis may result in the same reconstructed tract (primary direction unchanged) although the diffusion tensor is less ellipsoidal (reduced FA value).

White matter tractography is an alternative method to describe 3D patterns of WM connections [9, 21, 22]. The most commonly implemented methods are deterministic tractography approaches using the first eigenvector to estimate the trajectory of a white matter fiber. The analysis starts from a certain point or ROI and progresses voxel by voxel along the first eigenvector along the white matter fiber in order to estimate the anatomical trajectory of a bundle within the limits predefined parameters. The most important parameters are the minimum FA value (directivity of a voxel) and acceptable angle between the two first eigenvectors of two adjacent voxels. Alternatively, fibre tracts can be calculated that connect two or more predefined ROIs. The advantage of this method is the very high illustrative value of the resulting tracts. The disadvantages of this method include definition of one or multiple ROI and the resulting operator-dependency and dependence of the reconstructed “tracts” on tractography parameters. The sensitivity of tractography analysis may be lower compared to direct assessment of FA or other diffusion parameters, for example, in the domain of Alzheimer disease [23]. The authors found significant differences in FA and diffusivity in Alzheimer disease compared to controls yet no significant differences in deterministic tractography in the same regions. This can be explained by the fact that deterministic tractography uses the principal direction of the first eigenvector to calculate fiber tracts. Note that FA may decrease in a given disease, yet as long as the primary direction of the diffusion tensor remains unchanged, the resulting deterministic tractography remains unchanged (see Figure 2).

An additional problem of deterministic tractography is the difficulty to reconstruct trajectories across regions with crossing fibres. Possibilities to overcome this limitation include assessment of higher-order diffusion images such as Q-ball imaging [24] or diffusion spectrum imaging (DSI) [25]. The basic principle of these “second generation” diffusion techniques is the acquisition of higher resolution imaging with several diffusion directions within each voxels, thereby trying to overcome the crossing fiber problem. The resulting data acquisition time is however considerably longer than conventional DTI sequences with the resulting motion artifacts mainly in elder patients.

#### 3.2.2. Probabilistic Tractography

Other possibilities to overcome the crossing fiber problem are probabilistic tractography approaches [26]. In contrast to the deterministic tractography described above, probabilistic approaches do not follow a trajectory along the first eigenvector from voxel to voxel in a deterministic approach, yet calculate the probability with which two voxels or regions are connected. While such analyses may successfully overcome the crossing fiber problem, yet the resulting images are clearly less intuitive than those of the deterministic tractography.

### 3.3. TBSS: Tract-Based Spatial Statistics

A recent method was developed by Smith et al. [27] that projects all individual DTI parameters onto a group average white matter skeleton. The advantages of this method is the alignment of all subjects' FA images in order to create a “mean FA skeleton,” that is, a group average brain skeleton of the major WM tracts. This technique provides us with data of the whole brain and is operator-independent. The disadvantages include the multitude of required processing steps. Additionally, the resulting images are less intuitive than the deterministic tractography described above. Moreover, this technique is aimed for group level analysis but not analysis of individual patients.

## 4. Applications of DTI Analyses

In order to illustrate the relevance of this new technique in the exploration of the structural correlates of major psychiatric illnesses, we will focus the following chapters on DTI application in bipolar disorder and Alzheimer disease.

### 4.1. Bipolar Disorder

Bipolar disorder is a psychiatric illness affecting 1–3% of the population. It is characterized by alternating depressive and maniac phases. Several structural studies support the hypothesis of neurodevelopmental disruptions in early life implicated in the pathophysiology of bipolar disorder [28]. Different neurodevelopmental models suggest that deficits of maturational processes in adolescence in combination with early developmental changes result in psychiatric illness [29]. MRI imaging has provided us with valuable information concerning neuroanatomical abnormalities in bipolar patients during the past decades. Findings from structural and functional neuroimaging suggest that the basis of mood dysregulation results from disruptions along the frontocortical-striatal-thalamic circuits [30, 31]. Consistent with this hypothesis, a neuropathological study revealed reduced volumes of the left nucleus accumbens, bilateral external pallidum, and right putamen [32]. At the microscopic level, neuropathology revealed changes in glial density and neuronal abnormalities in the prefrontal and anterior cingulate cortex of patients with bipolar disorder [33, 34]. The majority of the structural brain research of bipolar disorder concerned gray matter. The role of fiber tracts interconnecting cortical and subcortical regions remains to be elucidated because their potential lesions could be implicated in brain dysfunctioning. White matter hyperintensities (WMH) on T2-weighted and fluid attenuated inversion recovery (FLAIR) images have been repeatedly reported during the last years in bipolar disorder. MRI signal hyperintensities in deep white matter were first described by Dupont et al., and their presence was associated with an increased number of hospitalizations [35]. Since then several scientists examined the prevalence of WMH in subjects with bipolar disorder. Most results show an increased prevalence in subjects with bipolar disorder [35–38]. White matter hyperintensities have been associated with cardiovascular risk factors such as hypertension [39] as well as advanced age [37, 40]. An MRI study of children and adolescents revealed a higher risk and severity of WM lesions in children with bipolar disorder located predominantly in the frontal lobes [41]. Lesions described in bipolar disorder were mostly located in the deep white matter [36, 37, 42], recent meta-analysis showed that patients with bipolar disorder had 2.5 times more deep white matter hyperintensities compared to controls [43]. WMH lack specificity having been associated with other illnesses and, thus, cannot be a specific marker for bipolar disorder.

There have been 19 published DTI studies to identify WM changes in subjects with bipolar disorder. Results are highly heterogeneous reporting decreased FA in frontal and prefrontal regions in adolescents, children [44, 45], and adults [46]. Other studies revealed increase in MD [47] and ADC [48] in prefrontal and frontal regions of adults. Regenold et al. was the first to report higher ADC [49] in 8 different ROIs of WM of a bipolar disorder population suggesting microstructural alterations in WM. A recent study by Versace et al. [50] demonstrated abnormal right versus left asymmetry in FA in BD subjects in the orbitomedial prefrontal white matter. DTI research of projection fibers showed reduced FA in the posterior [51] and anterior [52] limbs of the internal capsule in bipolar patients. Mahon et al. [53] first performed a voxel-wise analysis of FA to detect group level differences in FA between BD and control subjects, and then used the identified regions as seed regions for deterministic tractography. The reconstructed tracts included the pontine-crossing tract, corticospinal/corticopontine tracts, and thalamic radiation fibers, consistent with the concept that bipolar disorder implicates dysregulation of cortico-subcortical and cerebellar regions. This study revealed equally reduced FA in left cerebellum consistent with previous studies implicating cerebellar abnormalities in the model of bipolar disorder [28, 31, 54]. Increased FA of thalamic radiation was also described by Versace et al. [50], though a ROI, analysis by Sussmann et al. [52], showed decreased FA in the superior thalamic radiation fibers of patients. A small number of studies examined association fibers and found decreased FA in the uncinate fasciculus [50] connecting the frontal and temporal lobes. Higher or lower FAs were equally described in the superior longitudinal fasciculus [55, 56]. The corpus callosum which connects the two hemispheres was found to have a decreased FA in the rostrum and body of the corpus callosum [44, 57]. Under the assumption of increased cerebral structural abnormality during disease progression and ageing, we recently examined 19 euthymic elderly bipolar patients and 47 controls in a combined analysis of VBM and TBSS. This study found a significant decrease of FA in the ventral part of corpus callosum in patients with bipolar disorder. The VBM analysis of grey matter demonstrated a reduction of grey matter density in bipolar patients in the right anterior insula, the head of the caudate nucleus, nucleus accumbens, ventral putamen, and frontal orbital cortex as compared to controls. There was no significant group difference in TBSS data of bilateral uncinate fasciculus, anterior, and posterior cingulum. The WM alterations assessed using DTI in this study were more sensitive than changes in grey matter assessed using voxel-based morphometry (VBM) in bipolar disorder [8].

In summary, white matter alterations in BD are very heterogeneous, relating probably to different patient populations and data acquisition and analysis. Nevertheless, there is a trend towards impaired white matter integrity in BD in particular in frontal regions.

### 4.2. MCI and Alzheimer Disease

Alzheimer disease is the most common form of dementia. Mild cognitive impairment is characterized by memory complaints reported by the patient, preserved cognition, and autonomy in daily activities in life [58, 59]. Mild cognitive impairment has been classified to two subtypes: amnestic (memory deficits) and nonamnestic (other cognitive deficits) [58]. Patients with amnestic MCI are thought to present prodromal lesions of Alzheimer disease [60] and convert to Alzheimer over the years when compared to elderly population without cognitive decline [61, 62].

Alzheimer disease is characterized by the formation of extracellular neurofibrillary tangles and senile plaques. These lesions are found in the cognitively intact subjects as well [63–69], and it still remains unclear if their presence is part of the disease process or the ageing process. Most MR imaging studies have focused their interest on grey matter changes related with the AD. Additional to grey matter abnormalities such as diffuse cortical and hippocampal atrophy [70], white matter damage has been described in several neuropathological [71–73] and neuroimaging [74] studies. Several hypotheses have been proposed concerning the pathophysiology of white matter damage. The first hypothesis is the one of Wallerian degeneration occurring after neuronal loss meaning that white matter damage follows grey matter damage in the same regions [75]. A second hypothesis called retrogenesis suggests that the latest myelinated regions are the more vulnerable ones and that degeneration occurs in the reverse pattern of myelogenesis [76]. The third hypothesis involves vascular damage contribution to white matter pathology [77]. With respect to this last hypothesis, MRI white matter hyperintensities reflecting small vessel disease have been associated with AD. The cognitive impact of these lesions located in periventricular regions and deep white matter remains controversial [78, 79]. It has been suggested that their location is the key element of their implication in cognition [80].

DTI has been used to describe and understand white matter lesions in patients with Alzheimer disease and MCI, as well as normal aging. We will not review in detail DTI findings in normal elderly subjects [46, 81]. We will focus our interest on DTI findings of MCI and AD subjects and their correlation to underlying pathophysiological mechanisms. Numerous studies showed a heterogeneous pattern of changes in mean diffusivity and fractional anisotropy. Increased MD was reported in regions including frontal [82, 83] and temporal lobes [82–87], parahippocampal white matter [84, 88, 89], and the posterior cingulum [88–91]. Decreased FA was reported in the same regions by most scientists [84, 87, 89, 91–93] as well as in white matter tracts such as the superior longitudinal fasciculus [90]. FA differences between MCI and AD have been reported in temporal [92] and posterior cingulum regions [88] reflecting a more widespread WM pathology in AD. Interestingly, a recent study revealed that the decrease of FA in the posterior cingulum tract was associated with all four cognitive domains (memory, language, attention, and visual-spatial processing). This is in agreement with functional MRI studies proposing that posterior cingulate cortex functions as the main connectivity network during resting-state fMRI, with the posterior cingulate being a key structure in the default mode network [94].

The underlying pathophysiology of WM damage still remains under debate. Huang et al. [92] showed that patients with AD presented a pattern of reduced axial diffusivity and increased radial diffusivity in temporal lobe consistent with axonal loss damage and Wallerian degeneration. Similar observations were described by other research teams [82]. These findings are consistent with neuropathological loss of myelinated axons at histopathological studies of postmortem brains [95]. A DTI study of a mouse model revealed demyelination in corpus callosum and axonal loss in the other white matter tracts [96]. Retrogenesis has been supported by several authors as a possible degeneration pattern reflecting a vulnerability of late-myelinated regions in AD evolution [97, 98]. Naggara et al. investigated white matter damage with DTI, and their findings correspond to the retrogenesis hypothesis model with a decreased FA in the WM of temporal, frontal lobe, and the splenium [99]. Other published papers supported this hypothesis as well [100–102].

A study by Lee et al. tested the hypothesis of vascular factors' contribution to degeneration by focusing both on normal-appearing white matter and white matter hyperintensities in controls, MCI and AD subjects and by integrating etiologic contribution of vascular risk and degenerative processes in FA changes [103]. They found that AD subjects had a significantly lower FA in highly organized fibers. On the opposite, vascular risk factors had an impact on less organized fibers in both normal appearing WM and WMH. Decreased FA in WMH regions was not associated with vascular risk or diagnosis, implying that these lesions represent an extreme, diffuse lesional consequence of white matter. Similar to bipolar disorder discussed above, assessment of white matter changes using DTI is more sensitive than grey matter changes assessed by using VBM [97].

In summary, white matter changes can be readily assessed using DTI in MCI and Alzheimer disease, showing a widespread deterioration of multiple DTI bases parameters in widely distributed networks clearly exceeding hippocampus and parahippocampal regions. Recent investigations found evidence supporting both, the secondary Wallerian degeneration hypothesis following neuronal loss and retrogenesis hypothesis, suggesting that a combination of both mechanisms may be present in MCI and AD.

## 5. Discussion and Future Directions

Findings of DTI parameters remain rather heterogeneous and contradictory both in bipolar illness and Alzheimer disease. White matter changes assessed by DTI affect various regions in the brain, indicating that these diseases affect several cortical circuits consistent with the idea that neurons in a given cortical region are connected with axons in distributed networks. A number of recent investigations that simultaneously assessed grey matter (VBM) and white matter (DTI) in various neurodegenerative diseases consistently reported higher level of significance of group differences of DTI as compared to VBM analysis [8, 97, 104]. This suggests that the DTI assessment of white matter changes is more sensitive than VBM assessment of grey matter changes in neurodegenerative diseases. Future studies are needed to determine whether this is due to more pronounced disease-related changes in white matter or due to a higher sensitivity of the different methods which might more readily detect white matter changes due to, for example, higher signal to noise ratio of the data measurement.

Despite *in vivo* and *ex vivo* animal and human studies, the correlation of the different DTI parameters and pathological lesions of white matter diseases still needs further clarification. Human studies with DTI differ in sample characteristics, sample size, and techniques of data analysis. For example, brain regions including crossing fiber tracts, such as the rostral pons, revealed no (or marginal) changes in diffusion anisotropy, yet an important change in fiber orientation [105]. The need for MR correlations with neuropathology is imperative in the future to better understand and interpret changes in FA, MD, AD, and RD. *Ex vivo* images have a high quality resolution to detect structural changes. DTI use in postmortem brains is complicated by the fact that water diffusion features changes dramatically postmortem, especially after brain fixation. Sun et al. compared calculated axial and radial diffusivity in a retinal ischemia mouse model *in vivo* and *ex vivo* [106]. They found that *ex vivo* radial diffusivity is comparable to *in vivo* in the detection of myelin changes. Axial diffusivity changes were not present in *ex vivo* samples. The same group showed that changes of DTI parameters' sensitivity is due to the fixation of brain tissues rather than the delay between death and their fixation [107].

The use of prefixed brain tissues is an alternative to neuropathology. Schmierer et al. compared histological changes such as myelin content, axonal count, and gliosis with DTI measurements of MD and FA in unfixed postmortem multiple sclerosis brains. They found a decrease of FA and MD that correlated with myelin content and to a lesser degree with axonal count [108]. FA remains the most commonly used parameter representing white matter integrity. Klawiter et al. investigated axial and radial diffusivity correlation with histopathological findings in multiple sclerosis (MS) postmortem brains [109]. They revealed a sensitivity of radial diffusivity in the detection of demyelination, but no correlation between axial diffusivity and axonal loss. Despite these discrepancies, the use of axial and radial diffusivity can be an essential aid in the interpretation of FA changes in the future.

### 5.1. Group-Level versus Individual Classification Analyses

Most neuroimaging studies use group comparisons to explore the biological substrates of a disease. However, this type of comparison does not provide individual markers of clinical evolution. In the case of MCI, an individual prediction of conversion to AD would be of great interest since not all MCI patients evolve to AD.

 In a very recent study, Haller et al. [110] reported significant DTI differences between stable MCI versus progressive MCI subjects. They assessed neuropsychologically 35 controls and 67 MCI subjects among whom 40 were stable and 27 progressive. FA, MD, RD, and LD were measured using TBSS. FA was significantly higher in controls compared to MCI in a network involving the corpus callosum, right temporal and frontal pathways. No significant difference was found between stable versus progressive MCI. Support vector machines (SVMs) [111] have been recently used to provide us with individual risk scores concerning MCI conversion to AD. Haller et al. used TBSS preprocessed DTI FA data and subsequent individual SVM classification in MCI and controls. The accuracy of the individual classification of controls versus MCI was up to 91.4% and stable versus progressive MCI was over 97%. Their results suggest that one SVM classifier may be sufficient to discriminate stable versus progressive cases even if the neuropsychological profile of MCI subgroups is unknown at the time of SVM analysis.

## 6. Conclusions

DTI is an interesting noninvasive *in vivo* neuroimaging method to assess white matter. Despite the heterogeneity of the experimental data, the increasing application and development of DTI in central nervous system pathologies (such as bipolar disorder and Alzheimer disease) including the combination analysis of the tensor's parameters (axial, radial, mean diffusivity, and fractional anisotropy) may provide in the near future potential biomarkers for early and differential diagnosis of these conditions. Further basic research with animal models and postmortem brain tissues is required to establish a better comprehension of the correlations between DTI findings and microscopic changes in white matter.

## Abbreviations

AD: Alzheimer disease
ADC: Apparent diffusion coefficient
DSI: Diffusion spectrum imaging
DTI: Diffusion tensor imaging
CSF: Cerebrospinal fluid
FA: Fractional anisotropy
MCI: Mild cognitive impairment
MD: Mean diffusivity
MRI: Magnetic resonance imaging
RD: Radial diffusivity
SVM: Support vector machines
VBM: Voxel-based morphometry
WMH: White matter hyperintensities.
